# Supplementary material for: Gene-environment interaction in molar-incisor hypomineralization
Source: PLoS One. 2021 Jan 6;16(1):e0241898. doi: 10.1371/journal.pone.0241898 (PMC7787379; doi:10.1371/journal.pone.0241898)
Supplement: Attachment — Submitted filename: rebutal letter_PLoS One MIH.docx [file pone.0241898.s006.docx]

September 9, 2020

To: The Editor, PLoS One

Dear Dr. Cray Jr.:

after carefully reading yours and the reviewers’ comments, we decided to substantially revise and resubmit our work entitled “**Gene-environment interaction in molar-incisor hypomineralization** .” We believe we have addressed all the concerns and are hoping it is suitable for publication in your journal.

This work explores genomic variants associated with cleft lip and palate as potentially associated with MIH.

We are looking forward to seeing our revised work being well received. Below are point-by-point answers for the critiques and all changes are marked in yellow.

Sincerely,

Alexandre R. Vieira, D.D.S., M.S., PhD

Professor of Oral Biology, Pediatric Dentistry and Human Genetics Director of Clinical Research and Director of Student Research Department of Oral Biology

University of Pittsburgh School of Dental Medicine

412 Salk Pavilion

Pittsburgh, PA 15213

Office: 412-383-8972

FAX: 412-624-3080

E-mail: [arv11@pitt.edu](mailto:arv11@pitt.edu)

Point-by-point response to reviewers:

Reviewer #1: In this study, the authors analyzed causes of Molar Incisor Hypomineralization (MIH). The term “MIH” came into use 20 years ago, and causes of MIH are unknown. This reviewer thinks that the topic is very important for dentists. However, the following points should be clarified prior to further consideration of publication of the manuscript.

MIH is defined as a hypomineralization of systemic origin of one to four permanent first molars frequently associated with affected incisors by Weerheijm (2001).

Ref: Molar incisor hypomineralisation (MIH) Eur J Paediatr Dent. 2003;4(3):114-20.

The authors described that these enamel defects usually occur when there are disturbances during the mineralization or maturation stage of amelogenesis. The first molar is finished mineralization of crown by 3 years old. However, the authors concluded that environmental factors affecting children that were 3 years of age or older were also hypothesized to play a role in the disease etiology.

The authors also described that genes interacted and contributed to predisposition of MIH. If genetic factors cause hypomineralization, the disease is not MIH but amelogenesis imperfecta. Genetic abnormalities cause hypoplasia in all teeth rather than locally.

**RESPONSE: The definition cited above from Karin Weerheijm (2001) that the condition is o systemic origin does not exclude the possibility that it has a genetic component. We have suggested this for 8 years now and more and more scientists have surrendered to the idea at this point. We indeed believe MIH is possibly an extension of amelogenesis imperfecta, a localized one, at least in some instances. We added the opening paragraph of the Discussion section to address this reviewer’s concern. Further, we had originally included discussion in the paper regarding the time of mineralization of molars crowns and the variable “infection at 3 years of age.” It was on the original third paragraph of the Discussion section.**

Reviewer #2: The English language needs to be professionally revised. There are straightforward grammar issues. For example paragraph 4 in introduction “Risk factors for MIH did not fully explained” … and paragraph 5 in introduction “has been documented as responsible for not only affect amelogenesis…”. “The chance of the less common allele of TGFA rs930655 in addition to the IRF6 marker allele to increase the chance …”

**RESPONSE: We carefully revised the text for grammar and style and made the above pointed out corrections.**

Reviewer #3: This manuscript deals with an interesting topic and currently one of the forefront problems in the field of paediatric dentistry, i.e. MIH, focusing on its aetiology. However, there are some issues with this manuscript. In comments, only methodology and results of the study are addressed.

There is no information on how children were selected. Data on children’s age is missing.

**RESPONSE: This information was in the supplemental file. We moved if to the text.**

Who performed dental examinations? Were the dentists who performed dental examination calibrated?

**RESPONSE: This information was in the supplemental file. We moved if to the text.**


Regarding MIH diagnostic criteria; description of the dental examination results related to MIH affected teeth is essential (e.g. which teeth are MIH-affected, what was MIH severity).

**RESPONSE: This information was in the supplemental file. We moved if to the text. The original cohorts had originally assigned subjects are affected or not affected.**


Text describing environmental factors is very vague. How was the data on environmental factors obtained (e.g. medical history, a questioner)? Who answered the questions about environmental factors (e.g., children, parents, teachers,..)? Were all participants asked the same questions? On which environmental factors that could be associated with MIH were participants asked?

**RESPONSE: We added the information as requested. How the data was originally obtained (questionnaire) was original described in the methods.**

Why do the authors proceed from a hypothesis that environmental factors affecting 3 year-old children or older play a role in the aetiology of MIH? In MIH, the insult to the ameloblasts is likely to occur either prenatally or in the first year of life (Mangum and Farah).

**RESPONSE: We had originally added some Discussion regarding the variable of infection at 3 years of age. It is quite possible the disruption of amelogenesis happens later as well, not necessarily during the secretion phase only, but during the mineralization phase as well.**

Since the aim of the study was to clarify the aetiology of MIH, it is not clear why various potentially harmful aetiological factors would be considered as a single potential cause of MIH.

**RESPONSE: It is not the case we considered the various risk factors as a single potential cause. It is just that our analysis suggest one particular one associated.**

The results of the study did not show significant association between the selected SNPs and MIH. The study also does not provide solid evidence for the IRF6 and TGFA interaction, nor that the development of MIH is associated with any of these genes. Moreover, as the authors have already stated themselves, the size of each cohort is small for such research.

**RESPONSE: This is correct and it is clearly stated in the Results and Discussion sections. Suggestive results only come from the combined analyses of different factors.**

Figure 1: A description of each of the six images is missing.

**RESPONSE We added the description as requested.**

Reviewer #4: Thank you for the opportunity to review this manuscript, ‘Gene-environment interaction in Molar Incisor Hypomineralisation’. I congratulate the authors for undertaking very broad and comprehensive approach to investigating genetic interactions in MIH. However, due to two major concerns with this paper, I cannot support publication of this manuscript. My main concerns are:

(1) The choice of genetic variants – these appear to have been measured due to their relevance in a different condition (cleft lip and palate) which the authors suggest may be linked. However, adopting this candidate-based approach and then, by stratifying the data when significant results were not obtained so that a ‘statistically significant’ could be reported, seems to misrepresent the real outcome of the study. I suggest there are potentially more biological plausible interactions that could have been investigated that could be supported by existing evidence relating potential genetic and environmental risk factors. I note the authors do acknowledge some of the limitations however these major limitations do not seem to be considered when reporting the study conclusions.

**RESPONSE: We revised the conclusions to address this concern.**

(2) The observational component is particularly weak – participant recall of ‘medication use’ is prone to many biases and does not contribute to the existing evidence base regarding MIH. In order to investigate a complex causal relationship such as this, the authors needed to develop a detailed analysis plan with consideration of potential confounders etc.

**RESPONSE: This is an issue inherent to any observational study that includes recalling events many years prior. We added text in the last paragraph of the discussion to acknowledge this.**
