# Supplementary material for: Gene-environment interaction in molar-incisor hypomineralization
Source: PLoS One. 2021 Jan 6;16(1):e0241898. doi: 10.1371/journal.pone.0241898 (PMC7787379; doi:10.1371/journal.pone.0241898)
Supplement: Attachment — Submitted filename: rebutal letter2_PLoS One MIH.docx [file pone.0241898.s008.docx]

September 9, 2020

To: The Editor, PLoS One

Dear Dr. Cray Jr.:

after carefully reading yours and the reviewers’ comments, we decided to revise again and resubmit our work entitled “**Gene-environment interaction in molar-incisor hypomineralization** .” We believe we have addressed all the concerns and are hoping it is suitable for publication in your journal. The two most salient comments from one of the reviewers, the idea that MIH does not have a genetic component and the timing of mineralization of the first permanent first molar being at 1 year of age appeared to us not supported by the current knowledge. We addressed these concerns and added more discussion and believe those are a matter of opinion and we urge that PLoS let the readers react and decide how to interpret our work. Hopefully our work will motivate a change in direction for the field with less of an emphasis on association studies of elusive environmental factors and better designed gene-environmental model studies.

This work explores genomic variants associated with cleft lip and palate as potentially associated with MIH.

We are looking forward to seeing our revised work being well received. Below are point-by-point answers for the critiques and all changes are marked in yellow.

Sincerely,

Alexandre R. Vieira, D.D.S., M.S., PhD

Professor of Oral Biology, Pediatric Dentistry and Human Genetics Director of Clinical Research and Director of Student Research Department of Oral Biology

University of Pittsburgh School of Dental Medicine

412 Salk Pavilion

Pittsburgh, PA 15213

Office: 412-383-8972

FAX: 412-624-3080

E-mail: [arv11@pitt.edu](mailto:arv11@pitt.edu)

Point-by-point response to reviewers:

Reviewer #1: This author commented two problems of this manuscript. I couldn't get satisfying answers from authors.
The causes of disorders in amelogenesis can be divided into those caused by genes (genetic factors) and those not caused by genes (systemic causes, local causes). The former is hereditary disorder (amelogenesis imperfecta), which affects all teeth. The latter is enamel hypomineralization (enamel hypoplasia or enamel hypocalcification) caused by impaired ameloblast function. Enamel hypomineralization is chronological disturbance. MIH is included in enamel hypomineralization. MIH is a local enamel hypomineralization not amelogenesis imperfecta. Authors responded to my comments as that “MIH is possibility an extension of amelogenesis imperfecta”. The authors also have suggested that genetic abnormality is a one of the factors of MIH. I cannot believe this hypothesis. The disorder is not hypomineralization of enamel but amelogenesis imperfecta. “amelogenesis imperfecta” and “enamel hypomineralization” are often misunderstood as a single disorder and expressed in a term. However, they are actually different disorders. The former means hereditary disorders while the latter means nonhereditary congenital disorders considered as chronological disturbances. If enamel hypomineralization is caused by genetic abnormalities, enamel hypomineralization (MIH) is recognized in all teeth.

**RESPONSE: The assumption that a genetic condition affecting teeth would have to be present in al teeth is simply incorrect. We understand that is a misconception in the field and we added more discussion to the matter to address this concern. It is not a matter of opinion anymore, MIH fits well a condition with a complex mode of inheritance. Genetic conditions that have mendelian forms of inheritance are actually not the most typical examples of disease in human. Most common diseases in humans, such as cardiovascular diseases, diabetes, cancer (and dental caries, periodontitis, MIH) have complex modes of inheritance.**

This reviewer also commented on the timing of enamel mineralization. MIH is defined hypomineralization of enamel affecting affects one or more permanent first molars with or without permanent incisor involvement. Calcification of the first molar begins at birth, and crown formation (mineralization of enamel) completed at 30-36 months. The same opinion was found in reviewer #3.

**RESPONSE: As we discussed originally, most of the children included in the analysis had medications since their first year of life. We described evidence (original reference 19) that the mineralization of first permanent molars can be still occurring pass 36 months and up to 48 months. We added more discussion to highlight this concern.**

Reviewer #3 Why do the authors proceed from a hypothesis that environmental factors affecting 3 year-old children or older play a role in the aetiology of MIH? In MIH, the insult to the ameloblasts is likely to occur either prenatally or in the first year of life.
The authors responded that “It is quite possible the disruption of amelogenesis happens later as well, not necessarily during the secretion phase only, but during the mineralization phase as well.”
This reviewer thinks that the authors don’t consider mineralization phase of the first molar. Environmental factors affecting children that were 3 years of age or older never concern to MIH. This factor affects permanent teeth except for the first molars.

**RESPONSE: As we discussed originally, most of the children included in the analysis had medications since their first year of life. The impression that first molars are somehow immune from mineralization disturbances after the first year of life, knowing that mineralization of these teeth continues into 36 to 48 months is probably not current. We added more discussion to highlight this concern.**

Reviewer #2: After reviewing the revised submission and the response to the reviewer comments, I would like to inform the authors that NOT all my concerns were addressed. only the English language point was addressed!!

Please refer to the attached point by point review document (8 points in total) and provide point by point explanations and amendments within the body of the manuscript.

Throughout the manuscript

1. English language needs to be professionally revised. There are straightforward grammar issues. For example, paragraph 4 in introduction “Risk factors for MIH did not fully explained” … and paragraph 5 in introduction “has been documented as responsible for not only affect amelogenesis…”. “The chance of the less common allele of TGFA rs930655 in addition to the IRF6 marker allele to increase the chance …”

**RESPONSE: We made these corrections in the past version.**

2. A disorder is used commonly to refer to a group of conditions. The authors need to logically justify why they are using the word “disorder” to refer to MIH in many areas of the manuscript? Otherwise, the word disorder needs to be substituted throughout.

**RESPONSE: We revised the text as requested.**

Introduction 3. First paragraph, line 7: Did the authors omit the preposition “with” mistakenly in the sentence “usually permanent incisors”…??? This part of the sentence needs to be rephrased as the involvement of incisors is not always necessary to identify a case of MIH!

**RESPONSE: We revised the sentence as suggested.**

4. Third paragraph, line 17: “When full maturation is not complete at eruption, a post eruption maturation occurs through mineral ions from the saliva …” All newly eruptive enamel undergoes post eruptive mineralization regardless of the mineralization status of enamel. Please explain why you introduced “post-eruptive mineralization” here? Does it serve any purpose related to the objective of the study? if yes, what?

**RESPONSE: We deleted the sentence as suggested**

Methods 5. Were the subjects’ medication history collected at 3 years, after 3 years of age, or from birth to 3 years? There are many contradictory and confusing statements. In the beginning of the results section under environmental factors “any type of medications taken after three years of age (Table 4)…” , At the end of the same paragraph “..In both groups we found statistical evidence for an interaction of IRF6 and TGFA genotypes and medication intake at 3 years of age (Table 4)…” Other statements in the discussion section: “The variable medication intake after 3 years of age was created to capture children with history of medication intake from birth and past 3 years….” “another limitation we can point out is that we included only medication taken after 3 years of age…” In the method section: “..any type of medication intake between 3 years of age and the time the sample was collected…” These statements are very confusing and need to be explained? the authors need to be consistent when stating the one (or many) time point(s) where history of medications intake was collected. Please amend and maintain consistency throughout the manuscript.

**RESPONSE: We revised the text to make it consistent as suggested. The variable was created to captured use of medication from birth and passed 3 years of age.**

6. Referring to the previous point and acknowledging that the mineralization of the first permanent molar takes place from birth up to 3-4 years, why did the authors set up the cut off age to after 3 years to collect medication history? please give detailed explanation.

**RESPONSE: 3 years of age is likely easier to remember because the child has achieved a vocabulary of 200 words and most pf what she says can be understood. We added a comment in the discussion to address this concern.**

7. What was the reason behind the limited availability of the medication’s intake history to two cohorts out of the four cohorts?

**RESPONSE: This variable was not included in these original studies.**

8. Referring to the supplemental document, the subjects’ health data were collected from health records in the Rio de Janeiro, Brazil cohort, while structured data questionnaires were used to collect the same data from the Federal University of Paraná cohort. Explain the following issue: a. What do the authors think about the non- homogenous method of collecting medication history between cohorts (questionnaire in one cohort and medical records in another cohort)? this needs to be emphasized in the limitation paragraph in the discussion. How would the previous limitation affect the results of this study? Discussion § Paragraph 1, line 4: the author wanted to emphasize the geographical variations of MIH estimates, however, the generalizability of the declining trend between north and south MIH estimates needs to be addressed with caution!, first of all because the study includes cohorts from south America (Brazil) and the south European/Mediterranean regions. Second, this “claimed” trend is actually reversed when the south American MIH prevalence estimates is compared with the recently published north American MIH estimates (10% - 13% ). Thus, the statement needs to be rephrased to reflect the populations studied!

**RESPONSE: We added the suggested limitation on how the data were collected depending on the cohort. The best estimated frequency is Brazil is 13% as well and aligns of the figures from North America. We added a comment in the discussion to acknowledge this reviewer‘s comment.**
