# Supplementary material for: Gene-environment interaction in molar-incisor hypomineralization
Source: PLoS One. 2021 Jan 6;16(1):e0241898. doi: 10.1371/journal.pone.0241898 (PMC7787379; doi:10.1371/journal.pone.0241898)
Supplement: Attachment — Submitted filename: Review 2 PLOS response to the authors.docx [file pone.0241898.s009.docx]

1. The English language still needs to be revised. There are many grammar mistakes.

______________________________________________________________________________

2. The authors response to point 6 where the reviewer is questioning the cut off of 3 years to collect medication history is not satisfying nor convincing.

How is the child's vocabulary status a valid reason to choose this cut off age, when we know that the medication history is collected through direct questioning of the parent/caregiver?

______________________________________________________________________________

3. Many sentences need to be re-written in a third person narrative

This sentence is one example, but this style needs to be adopted through the manuscript.

"The fact that we did not always have detailed information about the type of medication the patients were taking due to self-reporting of this information forced us to include any type of medication intake in the

analysis. However, these were not vitamins or other supplements. But we are aware that this kind of observational study that aims to recover information from many years prior suffers from potential issues related to recall bias. For these reasons, we ask that our results are taken cautiously.”
