# Supplementary material for: Gene-environment interaction in molar-incisor hypomineralization
Source: PLoS One. 2021 Jan 6;16(1):e0241898. doi: 10.1371/journal.pone.0241898 (PMC7787379; doi:10.1371/journal.pone.0241898)
Supplement: Attachment — Submitted filename: rebutal letter3_PLoS One MIH.docx [file pone.0241898.s010.docx]

October 21, 2020

To: The Editor, PLoS One

Dear Dr. Cray Jr.:

after carefully reading yours and the reviewers’ comments, we decided to revise again and resubmit our work entitled “**Gene-environment interaction in molar-incisor hypomineralization**.” We believe we have addressed all the concerns and are hoping it is suitable for publication in your journal. The one most salient comment from one of the reviewers, the idea that the timing of mineralization of the first permanent first molar being at 1 year of age appeared to us not supported by the current knowledge. We addressed these concerns and added more detail and justification and believe those are a matter of opinion and we urge that PLoS let the readers react and decide how to interpret our work. Hopefully our work will motivate a change in direction for the field with less of an emphasis on association studies of elusive environmental factors and better designed gene-environmental model studies.

This work explores genomic variants associated with cleft lip and palate as potentially associated with MIH.

We are looking forward to seeing our revised work being well received. Below are point-by-point answers for the critiques and all changes are marked in yellow.

Sincerely,

Alexandre R. Vieira, D.D.S., M.S., PhD

Professor of Oral Biology, Pediatric Dentistry and Human Genetics Director of Clinical Research and Director of Student Research Department of Oral Biology

University of Pittsburgh School of Dental Medicine

412 Salk Pavilion

Pittsburgh, PA 15213

Office: 412-383-8972

FAX: 412-624-3080

E-mail: [arv11@pitt.edu](mailto:arv11@pitt.edu)

Point-by-point response to reviewer 2:

Reviewer #2: 1. The English language still needs to be revised. There are many grammar mistakes.

**RESPONSE: We carefully revised the text for grammar and typos. We did not find many grammar mistakes but corrected the ones identified.**

2. The authors response to point 6 where the reviewer is questioning the cut off of 3 years to collect medication history is not satisfying nor convincing.
How is the child's vocabulary status be a valid reason to choose this cut off age, when we know the the medication history is collected through direct questioning of the parent/caregiver?

**RESPONSE: We added more detail in the methods to address this concern. Evidence of cuspal enamel formation of first permanent molars can be detected past 3 years of age.**

**The comment of the vocabulary was in regard to recall bias, not enamel formation.**

3. Many sentences need to be re-written in a third person narrative
This sentence is one example but this style needs to be adopted through the manuscript.
"The fact that we did not always have detailed information about the type of medication the patients were taking due to self-reporting of this information forced us to include any type of medication intake in the
analysis. However, these were not vitamins or other supplements. But we are aware that this kind of observational study that aims to recover information from many years prior suffers from potential issues related to recall bias. For these reasons, we ask that our results are taken cautiously.”

**RESPONSE: We made the changes to adequate to the style the reviewer is requesting.**
