# Supplementary material for: Gene-environment interaction in molar-incisor hypomineralization
Source: PLoS One. 2021 Jan 6;16(1):e0241898. doi: 10.1371/journal.pone.0241898 (PMC7787379; doi:10.1371/journal.pone.0241898)
Supplement: Attachment — Submitted filename: PLOS ONE Reviewer comments.pdf [file pone.0241898.s005.pdf]

Throughout the manuscript

1. English language needs to be professionally revised. There are straightforward grammar issues. For example, paragraph 4 in introduction "Risk factors for MIH did not fully explained" … and paragraph 5 in introduction "has been documented as responsible for not only affect amelogenesis…". "The chance of the less common allele of *TGFA* rs930655 in addition to the *IRF6* marker allele to increase the chance …"

2. A disorder is used commonly to refer to a group of conditions. The authors need to logically justify why they are using the word "disorder" to refer to MIH in many areas of the manuscript? Otherwise, the word disorder needs to be substituted throughout.

Introduction

3. **First paragraph, line 7:**
   Did the authors omit the preposition "with" mistakenly in the sentence "usually permanent incisors"…???
   This part of the sentence needs to be rephrased as the involvement of incisors is not always necessary to identify a case of MIH!

4. **Third paragraph, line 17**: "When full maturation is not complete at eruption, a post eruption maturation occurs through mineral ions from the saliva …"
   All newly eruptive enamel undergoes post eruptive mineralization regardless of the mineralization status of enamel. Please explain why you introduced "post-eruptive mineralization" here?
   Does it serve any purpose related to the objective of the study? if yes, what?

Methods

5. Were the subjects' medication history collected at 3 years, after 3 years of age, or from birth to 3 years?
   There are many contradictory and confusing statements.
   In the beginning of the results section under environmental factors "any type of medications taken after three years of age (Table 4)…" ,
   At the end of the same paragraph "..In both groups we found statistical evidence for an interaction of *IRF6* and *TGFA* genotypes and medication intake at 3 years of age (Table 4)…"
   Other statements in the discussion section:
   "The variable medication intake after 3 years of age was created to capture children with history of medication intake from birth and past 3 years…."
   "another limitation we can point out is that we included only medication taken after 3 years of age…"

   In the method section: "..any type of medication intake between 3 years of age and the time the sample was collected…"

These statements are very confusing and need to be explained? the authors need to be consistent when stating the one (or many) time point(s) where history of medications intake was collected. Please amend and maintain consistency throughout the manuscript.

6. Referring to the previous point and acknowledging that the mineralization of the first permanent molar takes place from birth up to 3-4 years, why did the authors set up the cut off age to after 3 years to collect medication history? please give detailed explanation.

7. What was the reason behind the limited availability of the medication's intake history to two cohorts out of the four cohorts?

8. Referring to the supplemental document, the subjects' health data were collected from health records in the Rio de Janeiro, Brazil cohort, while structured data questionnaires were used to collect the same data from the Federal University of Paraná cohort. **Explain the following issue:**

a. What do the authors think about the non- homogenous method of collecting medication history between cohorts (questionnaire in one cohort and medical records in another cohort)? this needs to be emphasized in the limitation paragraph in the discussion. How would the previous limitation affect the results of this study?

Discussion

- **Paragraph 1, line 4**: the author wanted to emphasize the geographical variations of MIH estimates, however, the generalizability of the declining trend between north and south MIH estimates needs to be addressed with caution!, first of all because the study includes cohorts from south America (Brazil) and the south European/Mediterranean regions. Second, this "claimed" trend is actually **reversed** when the south American MIH prevalence estimates is compared with the recently published north American MIH estimates (10% - 13%). Thus, the statement needs to be rephrased to reflect the populations studied!
